# Significant difference in *Miscanthus* species root carbon exudation rate

**DOI:** 10.1093/aob/mcaf113

**Published:** 2025-06-02

**Authors:** Amanda J Holder, Rebecca Wilson, Karen Askew, Paul Robson

**Affiliations:** Institute of Biological, Environmental and Rural Sciences (IBERS), Aberystwyth University, Plas Gogerddan, Aberystwyth, Wales SY23 3EE, UK; Institute of Biological, Environmental and Rural Sciences (IBERS), Aberystwyth University, Plas Gogerddan, Aberystwyth, Wales SY23 3EE, UK; Institute of Biological, Environmental and Rural Sciences (IBERS), Aberystwyth University, Plas Gogerddan, Aberystwyth, Wales SY23 3EE, UK; Institute of Biological, Environmental and Rural Sciences (IBERS), Aberystwyth University, Plas Gogerddan, Aberystwyth, Wales SY23 3EE, UK

**Keywords:** *Miscanthus*, root exudates, soil carbon, root architecture, *Miscanthus sacchariflorus*, *Miscanthus sinensis*

## Abstract

**Background and Aims:**

The cultivation of *Miscanthus*, a giant perennial grass and promising biomass crop, is expected to increase globally in response to climate mitigation policies and sustainable agriculture goals. Little is known about root carbon (C) exudation and fine root architecture or how this might differ between *Miscanthus* species. To understand the functional biology of three diverse *Miscanthus* species and to evaluate impacts on soil C cycling, the aim of this study was to quantify root C exudation rates and track fine root growth.

**Methods:**

We use a controlled environment with plants grown in rhizotron boxes (28 L) to quantify living root C exudation rates and fine root growth of *Miscanthus sacchariflorus*, *M. sinensis* and *M.* × *giganteus*. Weekly non-destructive images of visible roots were analysed for root length density and root diameter during the growing season. Above- and below-ground biomass and C and nitrogen content were also recorded immediately after exudate sampling.

**Key Results:**

The exudation rate was significantly lower for *M. sacchariflorus* compared with *M. sinensis* and *M.* × *giganteus* (0.0 versus 0.6 g C g^−1^ root dry mass year^−1^). Coupled with this, *M. sacchariflorus* had greater above-ground biomass, a smaller increase in root mass and a higher root C concentration. Rapid root growth was observed, especially for *M.* × *giganteus*, for which root length density (0–30 cm depth) was higher compared with both *M. sacchariflorus* and *M. sinensis* in the earlier growth weeks.

**Conclusions:**

The results reveal a possible fundamental difference in nutrient resource acquisition and allocation between *M. sacchariflorus* versus *M. sinensis* and *M.* × *giganteus*. We estimate that *Miscanthus* root C exudation could add up to 2 g C kg^−1^ soil month^−1^ (during the peak growing season), a considerable influx of new labile C. This unique insight into differences in *Miscanthus* exudation indicates the potential for targeting *Miscanthus* breeding for enhanced soil C sequestration.

## Graphical Abstract

**Graphical Abstract:**
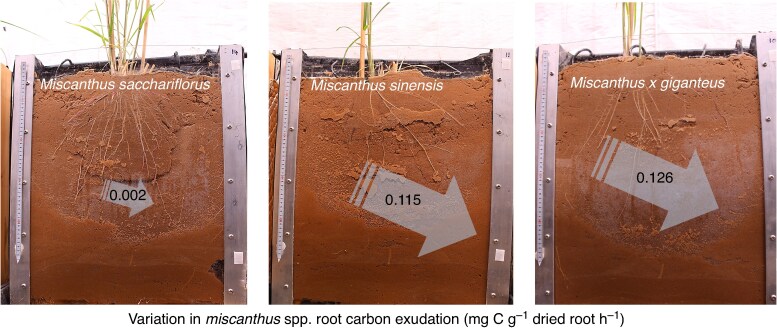

## INTRODUCTION

The cultivation of the biomass crop *Miscanthus* (a giant perennial grass) is expected to increase globally in response to climate mitigation policies (Calvin *et al.*, 2021; Clifton-Brown *et al.*, 2023). *Miscanthus* is an attractive crop owing to its rapid biomass growth, soil carbon (C) benefits and potential to achieve negative greenhouse gas emissions (e.g. if plant fixed C is embedded in products, increases soil C stocks or if emissions released during combustion are stored geologically through carbon capture and storage) (Robson *et al.*, 2019; Shepherd *et al.*, 2020; Iordan *et al.*, 2023). The protection and increase of soil organic carbon (SOC) sequestered in agricultural soils is vital in climate mitigation strategies and sustainable agriculture (Bossio *et al.*, 2020; Wiese *et al.*, 2021).

Plant root characteristics are a pivotal part of soil ecosystem processes. Different architectural (rooting depth and density), morphological (root diameter and specific root length), physiological (root respiration and exudation) and biotic (interactions with microorganisms) root traits of diverse plant species, interacting with various soil types, uniquely shape the rhizosphere (Bardgett *et al.*, 2014; Williams *et al.*, 2022). In particular, rhizodeposits of insoluble exudates of high molecular weight (e.g. border cells, mucilage) and soluble exudates of low molecular weight (e.g. sugars, amino acids) are at the interface of bi-directional soil–root interactions (Jones *et al.*, 2009). These root exudates provide several benefits to plants, including improved health and resilience, which, in turn, can enhance sustainable agricultural systems (Huang *et al.*, 2014) and play a key role in soil C cycling processes (Panchal *et al.*, 2022).

Root turnover and root exudates (along with inputs from the decomposition of above-ground plant residues) are primary pathways for atmospheric carbon dioxide (CO_2_), fixed through photosynthesis, to enter the soil C cycle (Kumar *et al.*, 2006; De Deyn *et al.*, 2008). Roots promote SOC stabilization through inputs of new labile C easily transformed into microbial products and subsequently sorbed onto soil minerals, and the formation of soil aggregates. Conversely, roots also contribute to SOC destabilization through: new inputs stimulating microbial activity that promote the use of existing C (known as priming; Kuzyakov *et al.*, 2000); organic acids chemically freeing previously bound C; and the destruction of aggregates exposing C to decomposition (Dijkstra *et al.*, 2021). Depending on exudate composition and soil conditions, root exudation can have contrasting effects for SOC sequestration, in some circumstances facilitating the accumulation of SOC and in others accelerating SOC loss (Wen *et al.*, 2022*a*).

Although part of the plant fixed C allocated to roots is released as CO_2_ from root and microbial respiration (Freschet *et al.*, 2021), root turnover and exudates from *Miscanthus* are estimated to account for ∼68 % of soil C input (Bertola *et al.*, 2024). However, owing to a lack of empirical data, SOC models generally rely on allometric functions of C inputs from *Miscanthus* roots, e.g. using functions relating to soil nitrogen (N) levels and root biomass (Juice *et al.*, 2022) or equations based on above-ground harvest yields and estimations of below-ground biomass (Bertola *et al.*, 2024).

Root exudates also promote plant acquisition of nutrients through the recruitment of beneficial microbes and the supply of fresh C, encouraging microbial growth and the subsequent mineralization of organic N and availability of soluble phosphorus (P) and iron (Fe) (Pantigoso *et al.*, 2022). It has been proposed that plants with thinner roots effectively search the soil for nutrients, whereas plants with thicker roots use symbiotic relationships with mycorrhizal fungi to a greater extent (Bergmann *et al.*, 2020; Wen *et al.*, 2022*b*). It has been found that plants can also adjust the chemistry of exudates to aid in adapting to various rhizosphere conditions and biotic and abiotic stresses (Sun *et al.*, 2021; Ahlawat *et al.*, 2024). Root exudation therefore drives the composition and function of soil microbial communities, regulates soil biogeochemical cycles, and can modify soil structure.

Additionally, the C supplied from plants to the soil via root exudates is also influenced by root traits. For example, exudation from fine roots (≤2 mm) is generally higher than from thicker coarse roots (>2 mm), and therefore the large pulses from smaller-diameter roots are likely to travel further into the soil (Finzi *et al.*, 2015). Deeper-rooting plants have the potential to impact SOC at lower soil depths, where exudates of similar composition can affect SOC in different ways owing to differences in soil conditions (de Graaff *et al.*, 2014; Lei *et al.*, 2023).

Specific root length (SRL; the length of root per unit of dry mass) is an indicator of the potential nutrient acquisition area per cost of biomass and is associated with root lifespan and decomposition rate (Freschet *et al.*, 2021). The relationship between SRL and decomposition varies from being positive for grasses to non-significant or negative for herbaceous and woody plants (Poirier *et al.*, 2018). Variation in component traits of root tissue density (RTD; the root volume per unit of dry mass) and root diameter account for variation in SRL (Freschet *et al.*, 2021). Fine roots are characterized by a low RTD and high SRL, and the distribution of roots within fine root diameter classes indicates root morphological diversity (Liu *et al.*, 2018; Erktan *et al.*, 2023). Roots <1 mm in diameter encourage the formation of macroaggregates within the soil matrix, aiding the short-term (years to decades) occlusion of soil organic matter (SOM) and promoting stabilized SOM via microaggregation (Poirier *et al.*, 2018).

In addition to variation attributable to plant developmental stage, plant health, season and time of day (Badri and Vivanco, 2009; Chaparro *et al.*, 2013; Rathore *et al.*, 2023) root exudation can differ by and within plant species (Mönchgesang *et al.*, 2016; Gao *et al.*, 2024). Therefore, it is important to understand the rhizosphere C inputs of novel crop types and the potential impact on C cycling.

The genus *Miscanthus* includes a number of species. The common commercial variety grown as a biomass crop for bioenergy and bioproducts (Brosse *et al.*, 2012; Arnoult and Brancourt-Hulmel, 2015; Moll *et al.*, 2020) is *Miscanthus* × *giganteus* (*M. × g*) (Greef and Deuter, 1993), with a potential commercial lifespan of ∼20 years (Winkler *et al.*, 2020). *Miscanthus* × *giganteus* is a natural hybrid of *M. sacchariflorus* and *M. sinensis* (Lewandowski *et al.*, 2000). Additionally, new crosses are being bred for improved resilience and harvest yields, capturing the benefits from this wide-ranging species (Awty-Carroll *et al.*, 2023).

Aboveground, *M. sinensis* species typically have multiple thin stems, whereas *M. sacchariflorus* are generally taller (∼2 m), with fewer and thicker stems (Robson *et al.*, 2013; Chae *et al.*, 2014). Underground, *Miscanthus* has a substantial root and rhizome system. *Miscanthus sinensis* species tend to have smaller rhizomes, with less of a creeping habit in comparison to *M. sacchariflorus*, which preferentially spreads laterally with chunky, thick-stemmed rhizomes (Chae *et al.*, 2014; Richter *et al.*, 2015). *Miscanthus* × *giganteus* is closer to the *M. sacchariflorus* form, with rhizome portions from established plants spreading into an area of ∼0.5 m^2^ once mature (∼3 years old) (McCalmont *et al.*, 2015).

As a rhizomatous plant, *Miscanthus* partitions C and nutrients from above-ground biomass to below-ground organs, sustaining growth and aiding with abiotic stress (Anderson *et al.*, 2011). The C:N ratios for *M. × g* roots and rhizome are in the region of ∼20 to ∼40 (Beuch *et al.*, 2000; Kahle *et al.*, 2001; Amougou *et al.*, 2011), although values have been observed to vary widely in *M. sacchariflorus* species (Poeplau *et al.*, 2019). In incubation experiments, *M. × g* roots are mineralized at a slower rate than rhizome, and this slower rate of decomposition could help to maintain SOC stocks (Beuch *et al.*, 2000; Amougou *et al.*, 2011; Ferrarini *et al.*, 2022).

Although deep rooting (≤2 m for *M. × g*; Neukirchen *et al.*, 1999), most of the below-ground biomass is found in the top 0.5 m of soil for all *Miscanthus* species (Hansen *et al.*, 2004; Richter *et al.*, 2015). In field conditions, RLD (in centimetres per centimetre cubed) for *M. × g* (6 years old) reduces with soil depth: 8–10 (0–10 cm depth); 4–6 (10–30 cm depth); 1–4 (30–60 cm depth); and 0–1 (60–100 cm depth) (Chimento and Amaducci, 2015; Gregory *et al.*, 2018). RLD for *M. sinensis* plants (of a similar age) also reduces with depth but was found to be higher throughout the profile: 12 (0–10 cm); 8 (10–30 cm); 3 (30–60 cm); and 2 (60–100 cm) (Gregory *et al.*, 2018). Mean root diameter for *M. × g* has been documented at 0.55 mm, in comparison to 0.47 mm for *M. sinensis* (Gregory *et al.*, 2018).

The knowledge base relating to root exudation and the fine root architecture of *Miscanthus* is small and relates predominantly to *M. × g*. Root C exudation rates have been recorded in the region of 2 mg C g^−1^ root dry mass (*M*_d_) day^−1^ for *M. × g* (Kaňova *et al.*, 2010; von Haden *et al.*, 2024), which could supply between 0.4 and 1.7 Mg C ha^−1^ year^−1^ (Agostini *et al.*, 2015) to the soil system. The exudates comprise assimilable organic C in the form of sugars, organic acids and amino acids, with the potential to increase soil heterotrophic respiration (Kaňova *et al.*, 2010; Hromádko *et al.*, 2010). However, in a comparison of annual and perennial crops (including *M. × g*) it was found that the lower exudation rate from perennials increased C and N mineralization from bulk soil organic matter, which might promote SOC sequestration over any losses from exudate priming (von Haden *et al.*, 2024). Differences in *Miscanthus* species root architecture and morphology conceivably impact on root exudation and turnover but, to our knowledge, there are no reports of root exudation rates for *Miscanthus* species other than *M. × g*. However, variation in SOC sequestration rates has been noted within *Miscanthus* species and hybrids (Zatta *et al.*, 2014; Gregory *et al.*, 2018; Chen *et al.*, 2020), and a positive relationship has been established between below-ground biomass and *Miscanthus*-derived SOC (Zatta *et al.*, 2014; Richter *et al.*, 2015).

Therefore, defining how *Miscanthus* species differ in terms of root exudation and fine root architecture and morphology is needed to understand the functional biology of this diverse species, to evaluate the potential impacts on SOC cycling and to inform modelled representations of plant carbon inputs. In this study, three *Miscanthus* genotypes were selected as representative of two broad *Miscanthus* species (*M. sacchariflorus* and *M. sinensis*) along with the hybrid, *M. × g*. The species were chosen because they are standard exemplar clones of parental species types of the commercial interspecific hybrid *M. × g*. A controlled environment experiment is used to address the following research questions and consider the potential implications for SOC sequestration:

1. Do *Miscanthus* species differentially affect the root exudation rate?
2. Are differences in root exudation explained by above- or below-ground biomass?
3. How do *Miscanthus* root growth traits differ between species?

## MATERIALS AND METHODS

The three *Miscanthus* genotypes used were *M. sacchariflorus* (*M. sac*), *M. sinensis* ‘*Goliath*’ (*M. sin*), and *M.* × *giganteus* (*M. × g*). The *M. sac* is a selection from wild-sourced Japanese *M. sacchariflorus* from Shikoku island and is a similar type to the *M. sacchariflorus* parent that created the standard *M. × g*. *Goliath* (*M. sinensis*, cv. *Goliath*) is officially origin unknown. It is likely to have been selected from an open pollination from a maternal tetraploid and a paternal diploid, both *M. sinensis*. *Miscanthus* × *giganteus* (Greef and Deuter, 1993; Stewart *et al.*, 2009), which is standard commercial *Miscanthus*, is an open pollinated hybrid of tetraploid *M. sacchariflorus* and *M. sinensis* (Linde-Laursen, 1993).

**Fig. 1.**
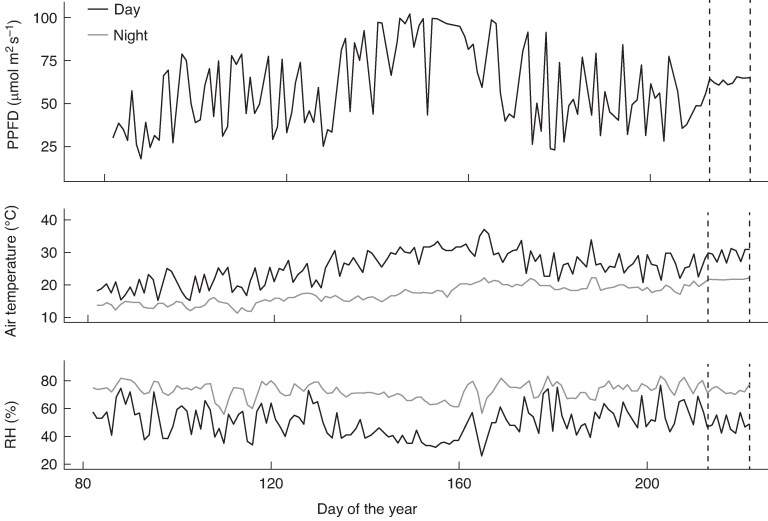
Glasshouse growing conditions from March to August: mean light [photosynthetic photon flux density (PPFD], air temperature (in degrees Celsius) and relative humidity (RH, as a percentage). Vertical dotted lines indicate the root exudate sampling period.

To obtain small plants of a comparable size, rhizomes from 10-year-old field grown *M. sac*, *M. sin* and *M. × g* were dug, split and pared back to one viable bud during early spring and planted into 1 L pots (using a John Innes No. 3 compost and perlite mix). Plants were grown on in a polytunnel until the next spring, when the senesced above-ground growth was cut and removed and the plants were gently washed from the pots. In March, five replicates of each species (*M. sac*, *M. sin* and *M. × g*) were transplanted into custom-made rhizotrons (root boxes constructed from a wooden frame and two sheets of 3-mm-thick Perspex, measuring 60  cm *×* 46 cm *×* 10 cm, ∼28 L; Supplementary Data Fig. S1). Prior to planting, the rhizotrons were filled with horticultural silver sand mixed with 250 g of a granular nutrient base fertilizer (5.5 N 7.5 P 10 K plus trace elements; Vitax Ltd, Coalville, UK). A further 10 g of fertilizer (mixed with sand) was added in June. The Perspex sides were covered with black plastic sheeting and surrounded with a layer of reflective insulation material. Rhizotrons were arranged in a random layout within a controlled-environment glasshouse setting, with natural light from above and growing conditions representing a temperate climate (Fig. 1). Rhizotrons were equipped with an automatic dripper watering system, with additional top-up watering done by hand, according to need. In mid-May, three *M. sin* and four *M. × g* dead plants were replaced with spares.

### Root exudate collection and analysis

Root exudates were sampled at the beginning of August from each of the plants, using a method based on and adapted from Phillips *et al.* (2008) and Sun *et al.* (2017). Rhizotrons were tipped at an angle of ∼60° and one side of the rhizotron Perspex was removed. Intact root portions were carefully extracted and cleaned with the aid of a wash bottle containing ultrapure water (PURELAB Classic, ELGA, UK). Root portions remained attached to the living plants throughout the sampling process.

The root portions were placed inside a 30 mL incubation tube (syringe) containing a small mesh circle (mesh size 0.8 mm × 0.8 mm, polyethylene) to retain ∼5 mL of new 1.0–1.3 mm glass beads (from falling into the tube outlet). The incubation tube was attached to a 40 mL collection vial using a length of tubing and a needle (Supplementary Data Fig. S2). After backfilling the incubation tube with beads, 10 mL of a C-free nutrient solution was added [0.5 mm NH_4_NO_3_ (ammonium nitrate), 0.1 mm KH_2_PO_4_ (potassium dihydrogen phosphate), 0.2 mm K_2_SO_4_ (potassium sulphate), 0.2 mm MgSO_4_ (magnesium sulphate) and 0.3 mm CaCl_2_ (calcium chloride); Phillips *et al.*, 2008]. The incubation tube was then sealed using a slit synthetic rubber stopper and parafilm. After securing the incubation tube to the rhizotron (avoiding injury/strain to the roots), the syringe, collection vial and rhizotron were covered with damp cloths and aluminium foil. The rhizotron was then covered with a sheet of black plastic and the roots incubated for 24 h (to allow for diurnal changes in exudation rates). Two root portions were incubated per plant (the average of the two used to provide one value per rhizotron). On each sampling occasion, three blanks were also incubated.

Following the incubation period, a second syringe was inserted into the collection vial and used as a pump to pull the liquid sample from the incubation tube. Two measures of 10 mL C-free nutrient solution were added to the incubation tube and pulled through to the collection vial. Then the solution in the collection vial was immediately transferred, through a 0.22 µm syringe filter, to a sample storage vial and kept cool until placed in a freezer (−20 °C) for storage until analysis. After retrieval of the solute sample, the incubated root portions were cut from the plant and measured for length. The root portions were then oven dried (60 °C) and weighed to obtain dry mass (*M*_d_). The RTD (using root dry matter content, according to Birouste *et al.*, 2014) and SRL were calculated for the incubated root portions using eqns (1) and (2).


(1) $$\mathrm{RTD}=\;\frac{\mathrm{root}\;\mathrm{dry}\;\mathrm{mass}}{\mathrm{root}\;\mathrm{fresh}\;\mathrm{mass}}$$

(2) $$\mathrm{SRL}=\;\frac{\mathrm{root}\;\mathrm{length}}{\mathrm{root}\;\mathrm{dry}\;\mathrm{mass}}$$

The total organic C (TOC) content of the solute samples was analysed using an Aurora 1030W TOC Analyser (OI Analytical, TX, USA). The mean TOC content of the blanks was subtracted from the TOC content for each sample prior to calculating the hourly root exudation rate (eqn 3). In a few instances, the blank C value was higher than the root sample C value, hence TOC was set to zero (von Haden *et al.*, 2024).


(3) $$\mathrm{Exudation}\;\mathrm{rate}\;(\mathrm{mg}\;C\;g^{-1}h^{-1})\;=\;\frac{\mathrm{TOC}\;(\mathrm{mg})/\mathrm{root}\;\mathrm{dry}\;\mathrm{mass}\;(g)\;}{\mathrm{incubation}\;\mathrm{time}\;(h)}$$

The yearly root exudation rate (in grams of carbon per gram root dry mass per year) was calculated in a similar way, using a period of 210 days to represent the full growing season (Agostini *et al.*, 2015).

### Above- and below-ground biomass

Immediately following the exudate sampling, the above-ground biomass was cut off at 2.5 cm above the sand surface, and the remainder of the below-ground biomass was washed and divided into root and rhizome. The root mass was separated further into new (white/red) and old (brown) root by colour. Above- and below-ground biomass was then oven dried (60 °C) and the fresh (*M*_f_) and *M*_d_ weights were recorded.

### C and N analysis

C and N analysis was carried out on the dried and ball-milled (Labman automated preparation system) new roots and above-ground leaves using an ANCA-SL elemental analyser (Sercon Ltd, Crewe, UK).

### Determination of root growth

For the duration of the study, non-destructive root growth and diameter were tracked weekly by removing the rhizotron outer coverings, placing a camera in a marked position and (using the same camera zoom settings) photographing the roots visible on one side of the Perspex. The digital photographs were then analysed for root length and diameter in 10 cm depth increments (0–10, 10–20, 20–30, 30–40, 40–50 and 50–60 cm) using the SmartRoot plug-in program (Lobet *et al.*, 2011) for ImageJ software (v.53; Rasband, 2022). Examples of roots traced using the SmartRoot plug-in are shown in the Supplementary Data (Figures S6-S8). Root length density (RLD, in centimetres per centimetre squared) was calculated as visible root length divided by image frame area. The variation in root diameter was classified by the percentage root length observed within nine diameter classes (in millimetres: 0–0.1, 0.1–0.2, 0.2–0.3, 0.3–0.4, 0.4–0.5, 0.5–1, 1–1.5, 1.5–2 and 2+), in addition to the percentage above and below 0.5 mm (Liu *et al.,* 2018). Concurrent measurements of above-ground growth were also taken (number of stems and height of the tallest stem to the top ligule).

### Statistical analysis

All ‘±’ errors reported are the s.e.m. Data analysis was carried out in R v.4.2.3 (R Core Team, 2023). Differences in the exudation rate and biomass data (as shown in Table 1) were explored using one-way ANOVAs and Tukey’s HSD *post hoc* tests (package ‘multcomp’; Hothorn *et al.*, 2008). The exudate data were log-transformed to improve normality of the model residuals.

**Table 1.** Mean values for biomass traits from the three *Miscanthus* species (*M. sac*, *M. sin* and *M. × g*) grown in the rhizotrons from March to August (*n* shows the number of observations, ±s.e., and different superscript letters denote statistical significance following Tukey’s *post hoc* tests; ns, not significant)

Species	*n*	No. of growth weeks	AG biomass	Stem height	No. stems	Leaf C %	Leaf C:N	Rhizome mass	Root mass (old)	Root mass (new)	Root C (new) %	Root (new) C:N	RTD	SRL
*M. sac*	5	19	18.34 ± 4.16^a^	106.3 ± 5.3^a^	6 ± 1.1^b^	42.5 ns ±0.4	19.5 ± 1.0^a^	16.01 ns ± 3.85	3.41 ± 1.00^b^	4.62 ns ±0.78	40.5±0.7^a^	46.7 ns ±2.3	0.19 ns ±0.04	857 ns ±162
*M. sin*	4	15	06.03 ± 1.98^b^	061.9 ± 13.8^b^	3 ± 1.0^a^	41.8 ns ±0.2	26.5 ± 2.4^b^	09.00 ns ± 2.16	0.49 ± 0.20^a^	2.93 ns ±0.27	32.2 ± 1.8^b^	39.2 ns ±6.0	0.10 ns ±0.00	1056 ns ±402
*M. × g*	5	13	10.74 ± 1.20^ab^	108.6 ± 8.4^a^	3 ± 0.3^a^	42.1 ns ±0.4	21.9 ± 2.5^ab^	09.00 ns ± 2.57	0.74 ± 0.15^a^	3.46 ns ±1.61	34.7 ± 1.6^b^	39.7 ns ±2.5	0.11 ns ±0.01	929 ns ±99

The mean growth weeks for plants in the rhizotrons differ owing to the replacement of dead plants. Above-ground (AG) biomass includes all stems and leaves (*M*_d_, in grams). Stem height (in centimetres) is the height of the tallest stem taken to the top ligule. The leaf carbon content (C %) and carbon-to-nitrogen ratio (C:N) relate to leaf subsamples taken from the AG biomass. Total plant rhizome mass and root mass (*M*_d_, in grams) relate to the biomass harvested after the collection of root exudates. New and old root mass was separated by appearance. Root C content (C %) and C:N relate to the new root portions. Root tissue density (RTD) and specific root length (SRL, in grams per centimetre) are described in the Materials and Methods and were calculated for the root portions incubated for the exudate sampling.

Growth weeks from 2 (i.e. 14 days since planting) to 11 were used to compare differences in RLD and root diameter (obtained from image analysis) over the experimental period. Owing to the replacement of dead plants, there were insufficient replicates in all species to compare by later growth weeks. RLD was analysed separately using one-way ANOVAs and Tukey’s HSD *post hoc* tests for each growth week and 10 cm depth segment. A square transformation was used with the 0–10 cm depth segment in growth week 2 to meet model assumption criteria. The percentage root length within each of the nine diameter classes was analysed using separate general linear mixed models (package ‘glmmTMB’; Brooks *et al.,* 2017) for each growth week, with species (*M. × g*, *M. sac* and *M. sin*) as the fixed factor and a beta distribution. Diameter classes above and below 0.5 mm were also explored using general linear models, with the fixed factors (and their interactions) of species and class (0–0.5 mm and 0.5–2+ mm) and a beta distribution. Results were summarized with ANOVA type 2 and type 3 sums of squares (package ‘car’; Fox and Weisberg, 2019), as appropriate, and Tukey’s HSD *post hoc* tests.

## RESULTS

### Root carbon exudation rates

**Fig. 2.**
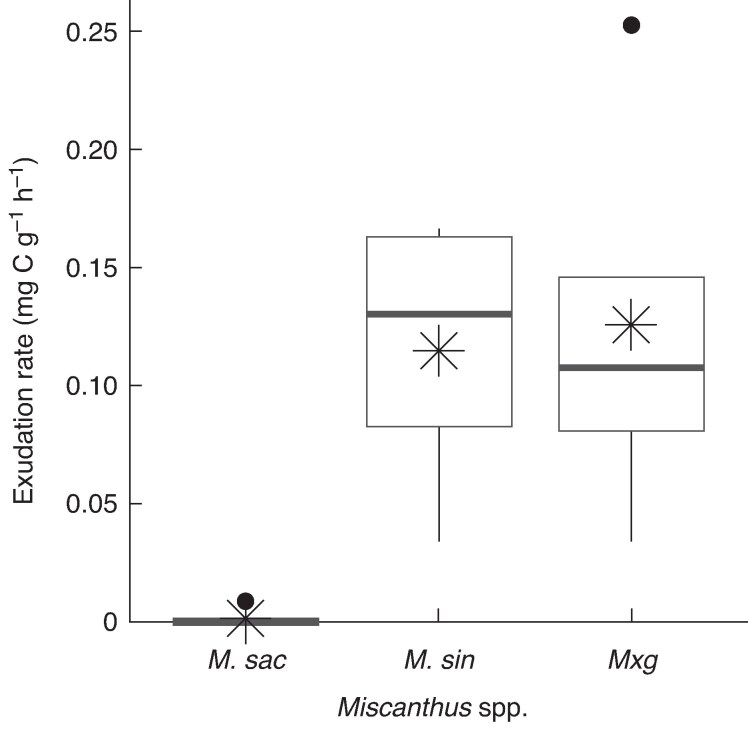
Hourly root exudation rates for each *Miscanthus* species (*M. sac*, *M. sin* and *M. × g*) grown in the rhizotrons, and calculated from living root portions incubated over a 24-h period. The asterisk denotes the sample mean (in milligrams of carbon per gram root dry mass per hour: 0.002, *n* = 5, *M. sac*; 0.115, *n* = 4, *M. sin*; 0.126, *n* = 5, *M. × g*), and the median is shown by the bar (0.000 *M. sac*; 0.131 *M. sin*; 0.108 *M. × g*).

There was a distinct variation between the C exudation from the *M. sac* roots, in contrast to *M. sin* and *M. × g.* The *M. sac* exudation rate (in milligrams of carbon per gram root dry matter per hour) of 0.002 ± 0.002 was significantly lower (*F*_2,11_ = 7.46, *P* < 0.01) than both *M. sin* (0.115 ± 0.031) and *M. × g* (0.126 ± 0.036) (Fig. 2). When calculated on a yearly basis (in grams of carbon per gram root dry matter per year, based on a 210-day growing season) exudation rates were 0.010 ± 0.010 (*M. sac*) 0.583 ± 0.158 (*M. sin*) and 0.636 ± 0.182 (*M. × g*). Root biomass for *M. × g* was higher than for *M. sin* (Table 1); therefore, whole-plant root C exudation from *M. × g* was ∼30 % higher compared with *M. sin* (2.67 versus 1.99 g C per plant year^−1^ for *M. × g* and *M. sin*, respectively). Whole-plant root C exudation was 0.08 g C per plant year^−1^ for *M. sac*.

### Above- and below-ground biomass

Despite a clear difference in the root C exuded between *M. sac* and the remaining two species, leaf C content was similar (Table 1). However, the C content of new roots was higher for *M. sac* compared with *M. sin* and *M. × g* (*F*_2,11_ = 9.63 *P* = 0.00; Table 1). There was no statistical difference in the root C:N ratio, but the leaf C:N ratio was higher for *M. sin* compared with *M. sac* (*F*_2,11_ = 4.40 *P* = 0.04), resulting from a lower leaf N content (Table 1). Stem height was significantly higher for *M. sac* and *M. × g* compared with *M. sin* at the end of the experimental period (*F*_2,11_ = 7.74, *P* = 0.00), and *M. sac* had a greater number of stems (*F*_2,11_ = 5.76, *P* = 0.02) (Table 1). Above-ground biomass was therefore greater for *M. sac* compared with *M. sin*, but not *M. × g* (*F*_2,11_ = 5.48, *P* = 0.04; Table 1). All the plants were within an establishment phase during the experiment, hence no flowering occurred.

There was no significant difference between the total root mass (old plus new) or the new root mass for each species. However, compared with the older root mass, new root mass increased by a higher percentage for *M. sin* and *M. × g* (35 % *M. sac*; 498 % *M. sin*; 368 % *M. × g*). Although total rhizome mass was generally higher for *M. sac*, variation between replicates meant that this was not significantly different from the other two species. At this young plant age, total below-ground biomass (rhizome and root) was greater than the total above-ground biomass (stems and leaves) for all three species. The total above- to below-ground biomass ratio was highest for *M. sac* and lowest for *M. sin* (0.8, 0.5 and 0.6 for *M. sac*, *M. sin* and *M. × g*, respectively).

### Root growth traits

**Fig. 3.**
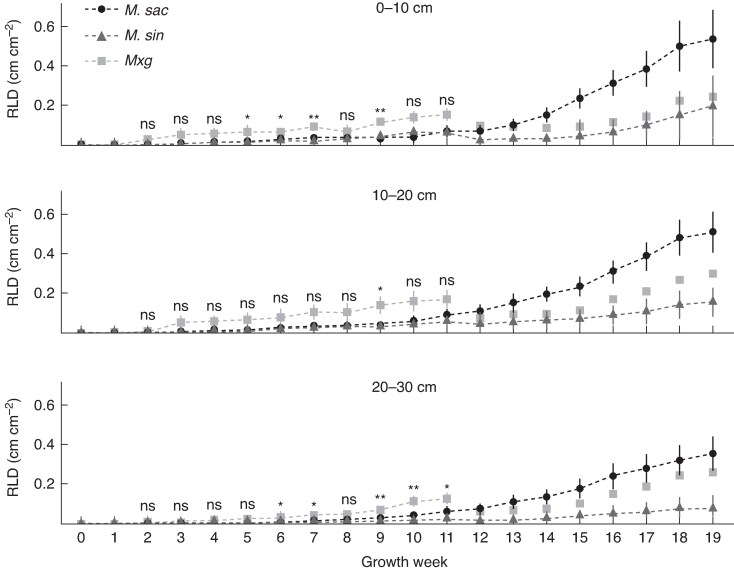
A comparison of mean root length density (RLD, as observed via the rhizotron) by growth week (number of weeks after planting) and depth segment (0–10, 10–20 and 20–30 cm). Error bars represent the s.e. and asterisks the significance level (*P < 0.05; **P < 0.01; ns, not significant). For *M. sac n* = 5, *M. sin n* = 4 and *M. × g n* = 5, for growth weeks 0–11. Owing to the replacement of dead plants, only one *M. × g* plant was measured for 19 weeks.

Analysis of the root images showed that roots were first visible in the lowest 50–60 cm depth segment for *M. × g* and *M. sin* plants in growth week 6, and for *M. sac* in growth week 7. Although there was no statistical difference in RLD in the visible roots below 30 cm depth, in the upper 0–30 cm segments RLD for *M. × g* was higher for a number of the earlier growth weeks (weeks 5, 6, 7 and 9 in the 0–10 cm segment; week 9 in the 10–20 cm segment; and weeks 6, 7, 9, 10 and 11 in the 20–30 cm segment) (Fig. 3). After growth week 11, higher RLD was observed for *M. sac* compared with *M. × g* and *M. sin*, but statistical analysis was not possible for the later growth weeks because only one *M. × g* plant survived the full 19 weeks of the experimental period.

**Fig. 4.**
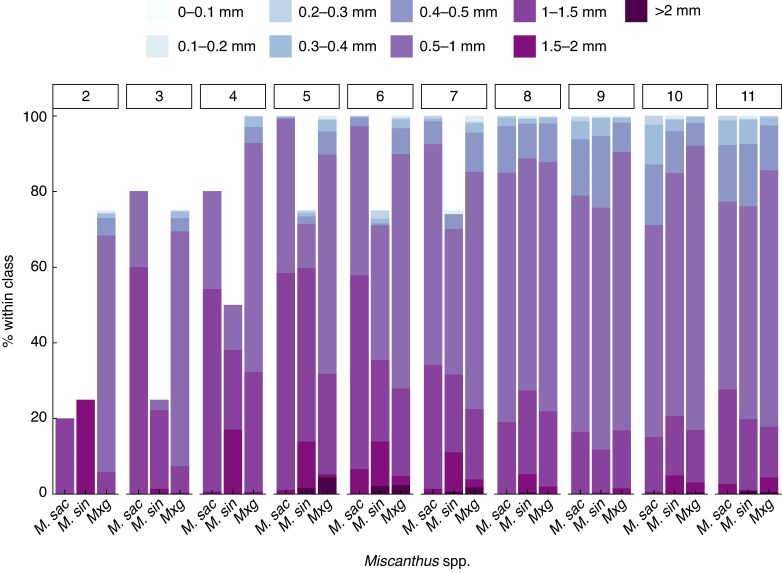
Mean percentage of visible root within nine diameter classes (in millimetres) for each species (*M. sac n* = 5, *M. sin n* = 4 and *M. × g n* = 5) and growth week (number of weeks after planting), based on image analysis.

It was observed during the exudate sampling that the *M. sin* roots were brittle and proved more difficult to extract than the *M. sac* and *M. × g* roots. *Miscanthus sacchariflorus* had the highest RTD and lowest SRL of the incubated root portions, but neither of these traits was significantly different by species (Table 1). Image analysis showed no discernible pattern of species differences in the percentage of visible root within each individual diameter class (Fig. 4). However, from growth weeks 3 to 11, the majority of roots were observed within classes above 0.5 mm [χ^2^(1) = 7.69, *P* < 0.01; χ^2^(1) = 8.61, *P* < 0.01; χ^2^(1) = 22.24, *P* < 0.001; χ^2^(1) = 22.66, *P* < 0.001; χ^2^(1) = 11.92, *P* < 0.001; χ^2^(1) = 49.25, *P* < 0.001; χ^2^(1) = 24.90, *P* < 0.001; χ^2^(1) = 21.20, *P* < 0.001; χ^2^(1) = 27.95, *P* < 0.001, for growth weeks 3–11, respectively], although only a small percentage of roots were observed above 1.5 mm (Fig. 4). In the root images, the thicker exploratory/nutrient transport roots were observed first, with thinner secondary branching roots increasing in later growth weeks (Fig. 4). All three species mainly exhibited a herringbone root structure with some dichotomous branching, and emerging *M. sac* rhizomes tended to spread laterally through the soil, whereas *M. sin* rhizomes formed closer together, in a clump (Supplementary Data Figs S3–S5).

## DISCUSSION

### Differences in root C exudation

In this study, we have shown that not all *Miscanthus* species are the same in terms of root C exudation. Although the recorded exudation rates (from live plants) were within the expected region (∼0.5 g C g^−1^ root *M*_d_ year^−1^, based on Kaňova *et al.*, 2010) there was significant variation in the rate for *M. sac* compared with *M. sin* and *M. × g* (0.0 versus 0.6 g C g^−1^ root *M*_d_ year^−1^). This suggests that *M. sac* might retain more C for internal use with a more conservative resource acquisition strategy (e.g. an efficient use of assimilated C and/or utilizing different symbiotic relationships with soil microorganisms). Other research has found the soil microbial community to differ for an *M. sacchariflorus* species compared with *M. sinensis* and *M. floridulus* species, whereby *M. sacchariflorus* had the lowest diversity of soil bacteria and fungi and the greatest difference in fungal community composition (Chen *et al.*, 2020).

The *M. × g* root C exudation rate recorded here was comparable to that of *M. sin*, leading to questions of the parental contribution to this physiological trait. Although in the hybrid it is assumed that the *M. sacchariflorus* genome is much larger and therefore will be likely to dominate the hybrid, phenotype studies have shown that, for example in Japan, *M. sinensis* and *M. sacchariflorus* grow in similar areas, and among the phenotypically *M. sacchariflorus* tetraploids, *M. sinensis* ancestry averaged 7 % and ranged from 1 to 39 % (Clark *et al.*, 2015). In terms of phenotype, several manuscripts have compared *Miscanthus* species with the *M.* × *giganteus* hybrid. There is no particular consensus regarding the dominance of the larger *M. sacchariflorus* genome. For example, the stem basal diameter (Robson *et al.*, 2013) and canopy senescence (Robson *et al.*, 2012) phenotypes of the hybrid lie between those of the two parental species groups. For other traits, there appears to be a transgressive segregation including tallest stem and dry matter yield, with the hybrid producing longer stems and higher yield (Robson *et al.*, 2013). In a study of cell wall chemistry, *M.* × *giganteus* contained significantly more cellulose and lignin and less hemicellulose compared with *M. sacchariflorus* or *M. sinensis* (Allison *et al.*, 2011). Differences were detected in how the cell wall components correlated with each other, suggesting that in *M.* × *giganteus* the cell wall is somehow regulated differently compared with *M. sinensis* and *M. sacchariflorus*. The complexity of trait associations is illustrated by an interesting study that appeared to show a seasonal switch in the biochemical response of the hybrid to temperature. *Miscanthus* × *giganteus* had intermediate behaviour, with characteristics of both chilling-tolerant and chilling-sensitive genotypes depending on seasonal timings, but sensitivity and tolerance were not linked to parental species groups in that study (Fonteyne *et al.*, 2018).

Exudation rates are likely to vary between and within growing seasons, depending on plant growth stage, age and other environmental factors, such as climate and soil conditions (Badri and Vivanco, 2009; Vives-Peris *et al.*, 2020). Exudate rates can also alter depending on soil or plant nutrient status (Jing *et al.*, 2023) and, given that sufficient nutrients were added to the growing medium, it might be that the *M. sac* rate was lower owing to a response to growing conditions not seen in *M. sin* and *M. × g*. However, to consider this possible effect, further investigation with different levels of N addition would be required.

The exudates in this experiment were measured from young plants with new roots, although the exudation rate for *M. × g* in this study (0.13 mg C g^−1^ h^−1^) was comparable to a previously recorded rate of 0.19 mg C g^−1^ h^−1^ obtained from 15-year-old field-grown plants (Kaňova *et al.*, 2010). However, the method of collection of root exudates differs between these two studies, and the slightly higher figure recorded by Kaňova *et al.* (2010) might be attributable to the sampling technique, where exudation was sampled using roots cut from the plant, potentially increasing the discharge of C compounds.

The root exudates were sampled at the point of peak plant growth for all the plants, and therefore the yearly exudation rate (calculated assuming the same rate for the whole growing season) provides an estimate at the top end of potential C inputs to the soil system. Exudation rates sampled from a field trial (on four occasions, and over 2 years, from 10-year-old *M. × g* plants) have been shown to differ greatly, whereby, although rates increased from July to August in both years, the scale of change varied between years, possibly reflecting differences in plant growth attributable to meteorological conditions (von Haden *et al.*, 2024). The two rates recorded by von Haden *et al.* (2024) during the month of August were 2.15 and 0.94 mg C g^−1^ root *M*_d_ day^−1^, both lower than the equivalent rate recorded in this study for *M. × g* (3.02 mg C g^−1^ root *M*_d_ day^−1^), where the plants were grown in optimal conditions.

### Variation in *Miscanthus* biomass and root growth traits

In light of the low *M. sac* exudation rate and in support of the possibility that *M. sac* was retaining C resources to support greater biomass growth, it was found that *M. sac* had a higher root C content compared with both *M. sin* and *M. × g*. *Miscanthus sac* also had the highest SRL and lowest RTD of the incubated root segments, which, although not significant, are traits that have been associated with low root exudation (Guyonnet *et al.*, 2018; Williams *et al.*, 2022).

Typically, in other grass species, there is a reliance on short-lived absorptive roots to facilitate responsive soil exploration and provide a greater surface area for nutrient uptake, whereas forbs and shrubs exhibit dichotomous branching, with several root orders comprising thin absorptive (in the range of 0 to ∼0.5 mm diameter) and thicker transport roots (between ∼0.25 and ∼1 mm diameter) (Bergmann *et al.*, 2020; Erktan *et al.*, 2023). The plants in this study contained both root types, with a dominance of roots between 0.5 and 1.5 mm. However, they represent developing root systems and, for each of the three species, although transport roots were visible in the rhizotron images first, this was followed by an increase in absorptive roots. A greater diversity of root diameter class and species difference can be expected as the plants mature (Gregory *et al.*, 2018) and can also alter in response to soil nutrient status (Erktan *et al.*, 2023). In accordance with the anticipated growth habit of established plants, emerging *M. sac* rhizomes spread laterally through the soil, whereas *M. sin* formed a denser clump. The lower stem height for *M. sin* also reflects the nature of the mature plant, where numerous shorter stems would be expected. The brittleness of *M. sin* roots was noted, which might indicate a shorter lifespan or might be a result of growing conditions (e.g. root zone moisture levels). However, the rhizotrons were automatically watered to keep the growing medium (sand) moist, and top-up watering was carried out manually only when required.

Rapid root growth was observed for all three species, with roots visible in the lowest rhizotron depth of 50–60 cm around growth week 6, and by the end of the study the root growth of most of the plants was restricted by the size of the rhizotron (60 cm *×* 46 cm *×* 10 cm). RLD was highest for *M. × g* in the 0–30 cm depth in the early growth weeks, which might have been impacted by the warmer air temperatures experienced by the four plants replaced in May compared with those planted in mid-March. However, rapid development of *M. × g* roots has also been observed with young field-grown plants (Black *et al.*, 2017; Holder *et al.*, 2025), and three *M. sin* plants were also replaced at the same time. New root mass increased more for *M. sin* and *M. × g*, whereas the higher above-ground biomass for *M. sac* suggests a concentration of resources on above-ground growth at this stage.

### Implications for soil organic carbon sequestration

Similar to an estimated C input from grass pasture root exudation (0.1–5 g C kg^−1^ soil month^−1^, *Lolium perenne*; Jones *et al.*, 2009), the exudation rate of 3 mg C g^−1^ root *M*_d_ day^−1^ (*M. sin* and *M. × g*) recorded in this study could potentially add 0.1–2 g C kg^−1^ soil month^−1^ to the soil system during the peak growing season [based on root biomass of 5–21 Mg ha^−1^ (Monti and Zatta, 2009; Martani *et al.*, 2021) and calculated to a soil depth of 0.6 m using an average soil bulk density of 1.4 g cm^3^]. An estimated 20 % of total soil CO_2_ efflux is derived from heterotrophic respiration of low-molecular-weight rhizodeposits (van Hees *et al.*, 2005). Therefore, calculated on an area basis and assuming a 20 % loss owing to respiration, the C input from *M. × g* exudates could be in the region of 2–10 Mg C ha^−1^ year^−1^ (for root biomass of 5–21 Mg ha^−1^ and based on a 210-day season), which should be taken as a peak estimate. In reality, owing to differences in field environmental conditions, this figure is likely to be lower. Based on the lowest (0.2 g C g^−1^ root *M*_d_ year^−1^) and highest (2.15 g C g^−1^ root *M*_d_ year^−1^) rate recorded from field-grown *M. × g* at a point in July and August (von Haden *et al.*, 2024), the figure for new C inputs could range from a low of 0.2–10 Mg C ha^−1^ year^−1^ to a high of 2–8 Mg C ha^−1^ year^−1^ (for root biomass of 5–21 Mg ha^−1^). However, the fate of this new C is subject to differing processes depending on soil biotic and abiotic conditions and, as a readily accessible form of new C, it might induce priming effects (Kuzyakov *et al.*, 2000; Wen *et al.*, 2022*a*). It is also worth noting that scaled estimates of C input from root exudation rely heavily on estimates of fine root mass where, in field conditions, it can be difficult to capture fully and to assess the extent of *Miscanthus* fine root systems.

Although root exudation is an important component of the soil carbon cycle, it is only one aspect of a complex system. In fact, despite the fundamental difference observed in the root C exudation rate for *M. sac*, this is not necessarily detrimental to the accumulation of SOC. In a long-term field trial, SOC stocks for these same three genotypes (at 10 years old) were 86, 85 and 79 Mg ha^−1^ for *M. sac*, *M. sin* and *M. × g*, respectively, and were not significantly different from each other (0–30 cm depth; A.J. Holder, IBERS, Aberystwyth University, UK, unpublished research). This could be attributable to species differences in the turnover of roots and rhizomes or inputs derived from above-ground litter and harvest residues. It is also likely that individual genotypic differences and not only broader species differences have an impact (Holder *et al.*, 2019). In other studies, soil organic matter and soil N were also reported to be significantly higher under *M. sacchariflorus* compared to *M. sinensis* (5-year-old plants; Chen *et al.*, 2020) and SOC under *M. sinensis* significantly higher than under *M. × g* (6-year-old plants; Gregory *et al.*, 2018).

The impact of *Miscanthus* fine root systems and exudation on SOC accumulation is still unclear. Roots <1 mm in diameter are generally thought to increase SOC by promoting soil macroaggregation (Poirier *et al.*, 2018), and the roots observed in this study were mostly <1.5 mm at this young growth stage, and it is likely that there would be an increase in the smaller root classes as the plants mature. The root trait of SRL can be an indicator of root lifespan, respiration and decomposition rate (Roumet *et al.*, 2016; Freschet *et al.*, 2021; Han and Zhu, 2021), but within this study we found no significant difference in SRL (although this might alter with plant maturity). The composition of root exudates coupled with soil conditions can have positive and/or negative effects on SOC sequestration (Panchal *et al.*, 2022), and it has been suggested that changes in root exudate quality (as opposed to quantity) can affect soil carbon cycling to a greater extent (Lei *et al.*, 2023). Exudate composition was outside the scope of this study but would be worthy of investigation.

### Conclusion

The root C exudation rate was significantly lower for *M. sac* compared with *M. sin* and *M. × g* (0.0 versus 0.6 g C g^−1^ root *M*_d_ year^−1^). Coupled with this, *M. sac* had greater above-ground biomass, a lower increase in root mass and a larger root C concentration. Therefore, the results of this study reveal what could be a fundamental difference in nutrient resource acquisition and allocation between *M. sac* versus *M. sin* and *M. × g* that might also influence soil microbial community composition. It is not certain whether the difference in exudation rate reflects a broader *Miscanthus* species difference or is related to the individual genotypes sampled. Further exploration of this finding (especially in different controlled-environment and natural field conditions) is desirable to unravel the connection between *Miscanthus* species physiochemical traits, exudation quantity and quality, and their role in shaping the rhizosphere microbial community and soil carbon cycling. This could unlock possibilities for targeting *Miscanthus* breeding for enhanced soil carbon sequestration. From this study, we estimate that *Miscanthus* root C exudation could add new C of ≤2 g C kg^−1^ soil month^−1^ (during the peak growing season).

## Supplementary Material

mcaf113_Supplementary_Data

## SUPPLEMENTARY DATA

Supplementary data are available at *Annals of Botany* online and consist of the following. Figure S1: (A) Custom-made rhizotrons: root boxes constructed from a wooden frame and two sheets of 3-mm-thick Perspex, measuring 60 cm × 46 cm × 10 cm, with a capacity of ∼28 L. (B) An example of a *M. sac* plant in rhizotron with visible roots (as at 31 July). Figure S2: (A) Extracted root portions are inserted into the incubation tube part-filled with glass beads. (B) Prior to the 24-h incubation period, root portions are sealed in the tube (backfilled with glass beads and C-free nutrient solution), and a collection vial is attached. During incubation, the tubes and exposed rhizotron face were covered with damp cloths, aluminium foil and a black plastic sheet. Figure S3: (A) One example of the full *M. sac* washed root system at the end of the study period (31 July), with laterally spreading rhizomes. (B) Zoomed-in portion of the first image (as highlighted by the red box). Figure S4: (A) One example of full *M. sin* washed root system at the end of the study period (31 July), with rhizomes forming into a clump. (B) Zoomed-in portion of the first image (as highlighted by the red box). Figure S5: (A) One example of full *M. × g* washed root system at the end of the study period (31 July), with less dense rhizome growth in compared with *M. sin* and less spread compared with *M. sac*. (B) Zoomed-in portion of the first image (as highlighted by the red box). Figure S6: one example of *M. sac* roots traced using the SmartRoot plug-in program for ImageJ (for clarity, in this image, line widths are not shown to scale) as at 31 July. Figure S7: one example of *M. sin* roots traced using the SmartRoot plug-in program for ImageJ (for clarity, in this image, line widths are not shown to scale) as at 31 July. Figure S8: One example of *M. × g* roots traced using the SmartRoot plug-in program for ImageJ (for clarity, in this image, line widths are not shown to scale) as at 31 July.
