# Supplementary material for: Significant difference in Miscanthus species root carbon exudation rate
Source: Ann Bot. 2025 Jun 2;136(3):553–65. doi: 10.1093/aob/mcaf113 (PMC12455722; doi:10.1093/aob/mcaf113)
Supplement: mcaf113_Supplementary_Data [file mcaf113_supplementary_data.pdf]

Supplementary information for:

“Significant difference in Miscanthus species root carbon exudation rate”

Amanda J Holder, Rebecca Wilson, Karen Askew, Paul Robson

a)

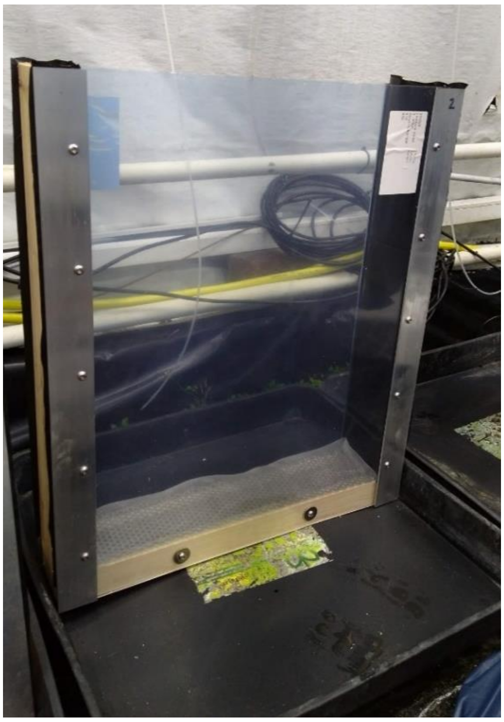

b)

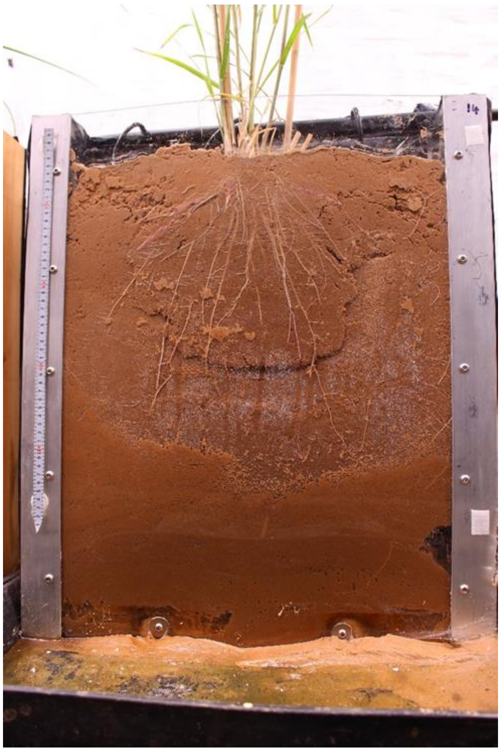

Figure S1 a) *Custom made rhizotrons: root boxes constructed from a wooden frame and two sheets of 3 mm thick Perspex, measuring 60 x 46 x 10 cm, with a capacity of ~28 litres* ***b)*** *An example of a M. sac plant in rhizotron with visible roots (as at 31 July).*

a)

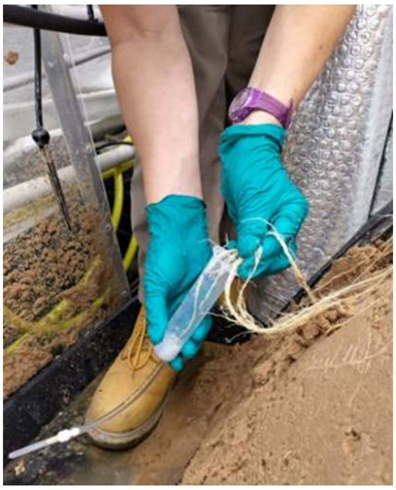

b)

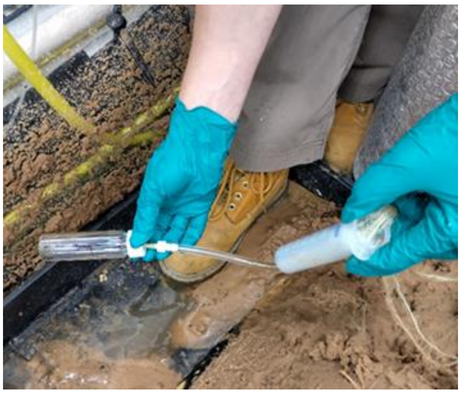

Figure S2 a) *Extracted root portions are inserted into the incubation tube part filled with glass beads;* ***b)*** *Prior to the 24-hour incubation period, root portions are sealed in the tube (backfilled with glass beads and C free nutrient solution) and a collection vial is attached. During incubation the tubes and exposed rhizotron face were covered with damp cloths, aluminium foil, and a black plastic sheet.*

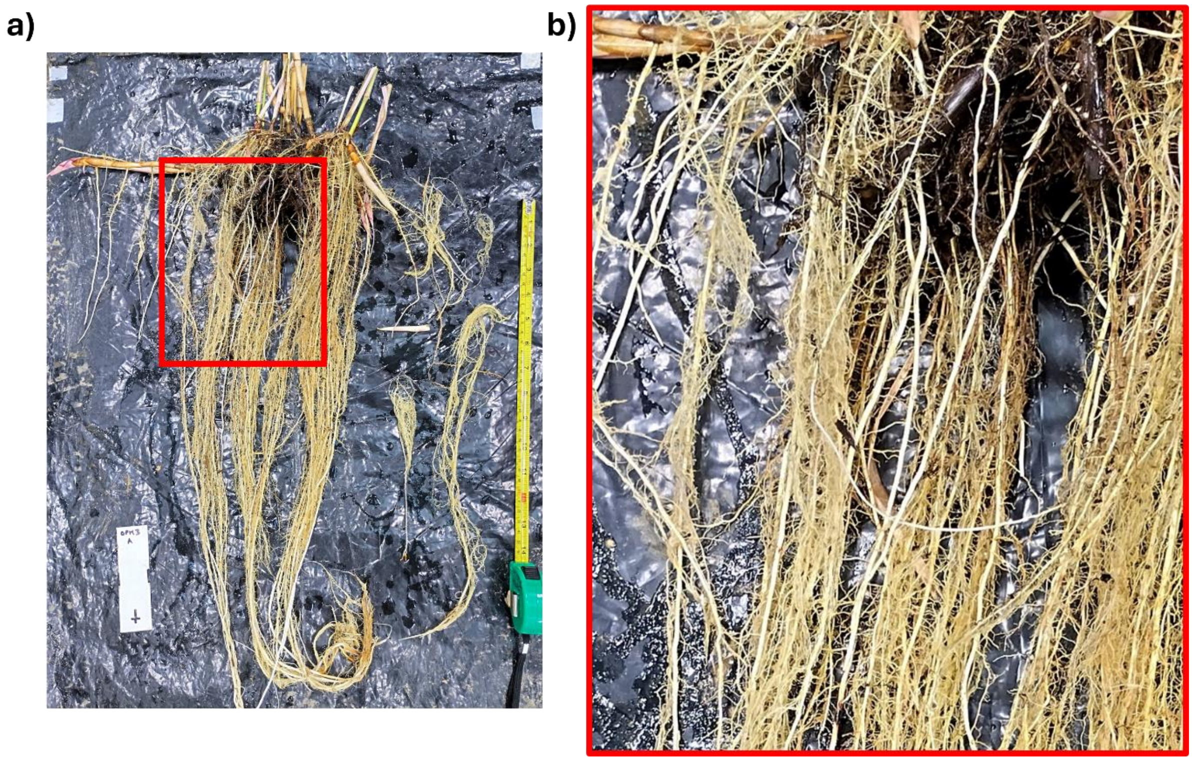

Figure S3 a) *One example of the full M. sac washed root system at the end of the study period (31 July) with laterally spreading rhizomes.* ***b)*** *Zoomed in portion of the first image (as highlighted by the red box).*

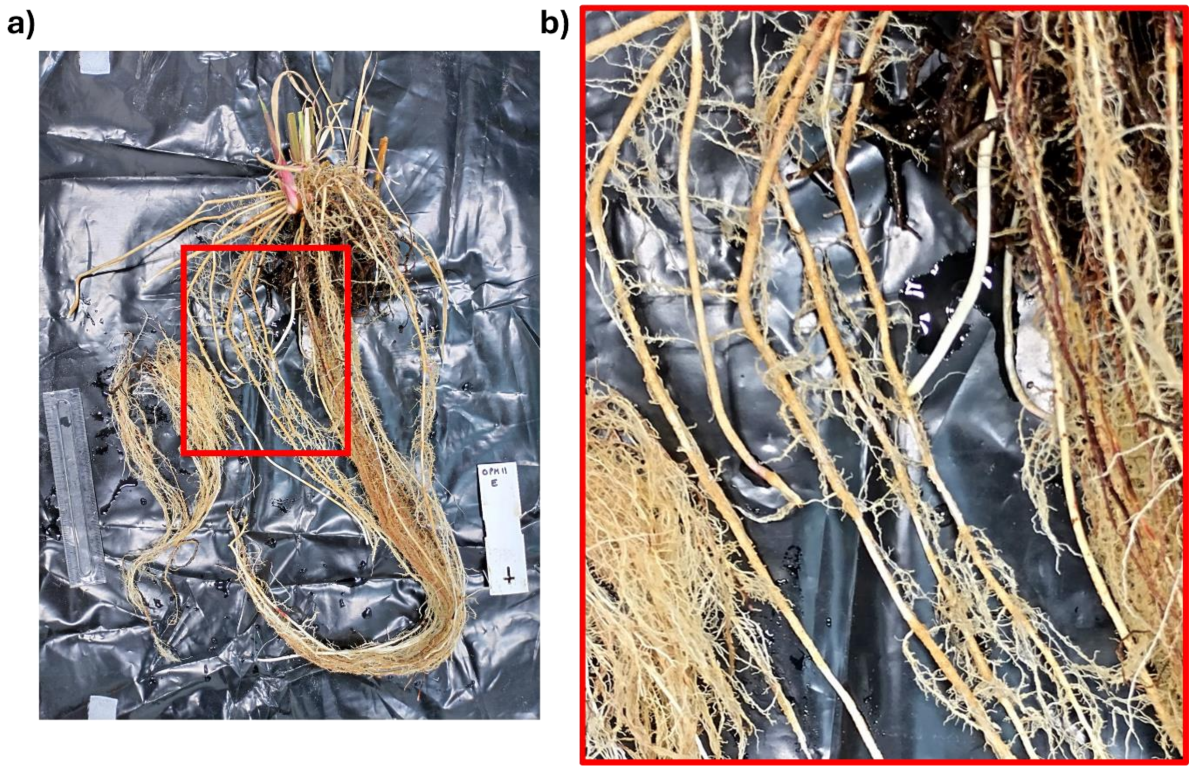

Figure S4 a) *One example of full M. sin washed root system at the end of the study period (31 July), with rhizomes forming into a clump.* ***b)*** *Zoomed in portion of the first image (as highlighted by the red box).*

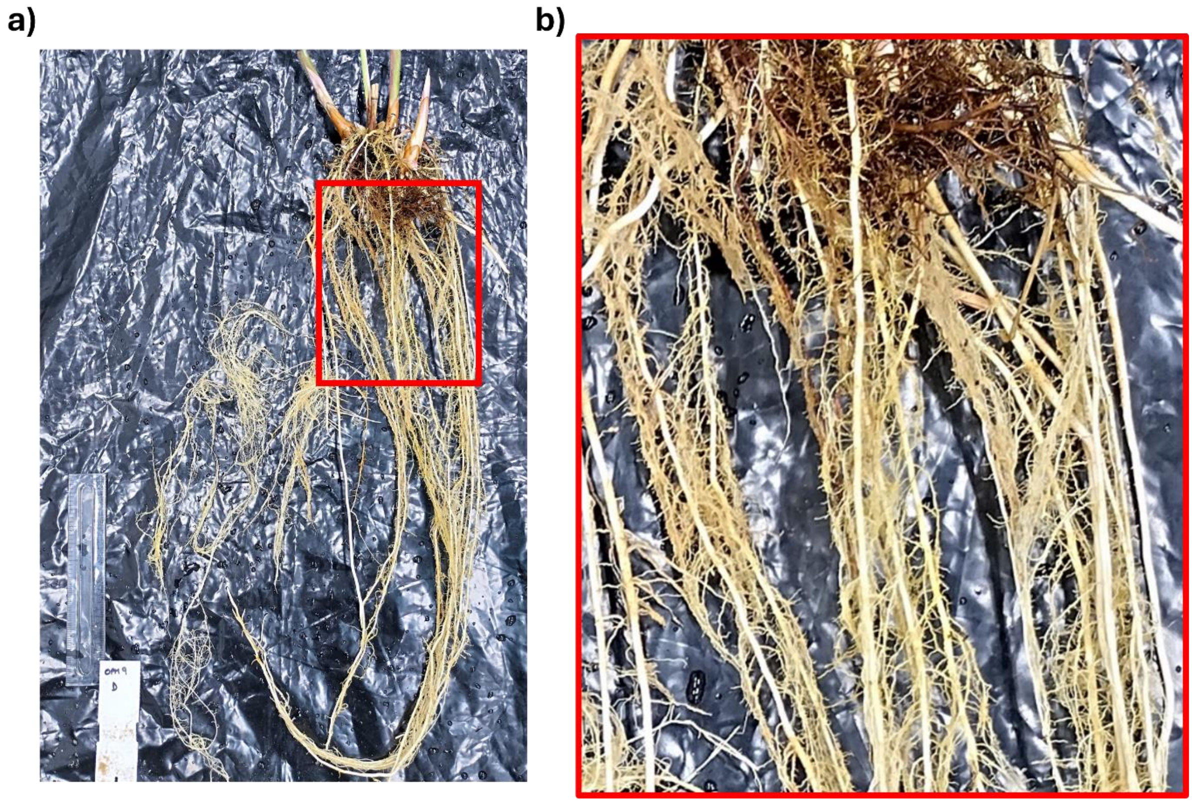

Figure S5 a) *One example of full M×g washed root system at the end of the study period (31 July), with less dense rhizome growth compared to M. sin, and less spread compared to M. sac.* ***b)*** *Zoomed in portion of the first image (as highlighted by the red box).*

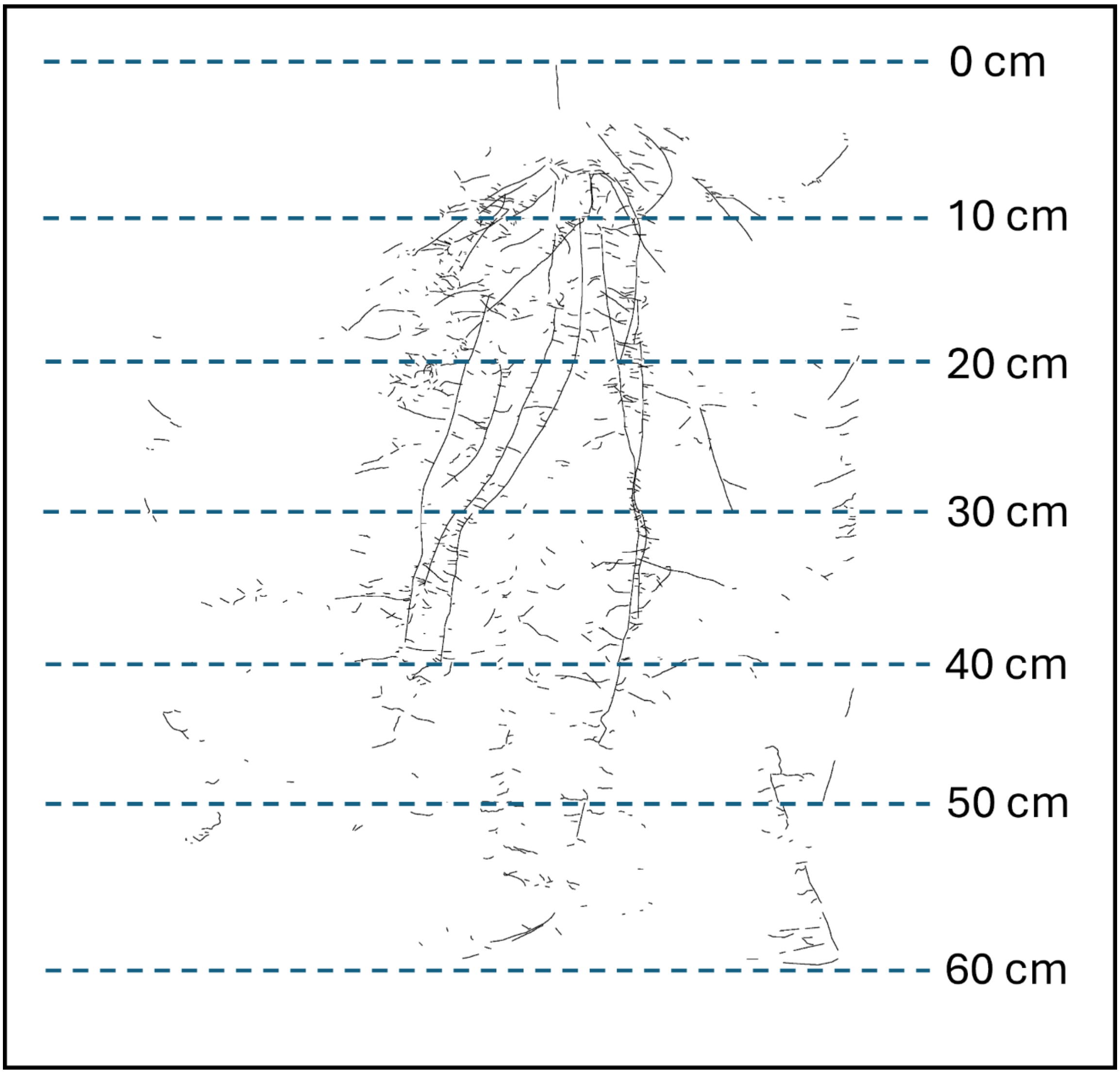

Figure S6 *One example of M. sac roots traced using the SmartRoot plug-in programme for ImageJ (for clarity, in this image, line widths are not shown to scale) as at 31 July.*

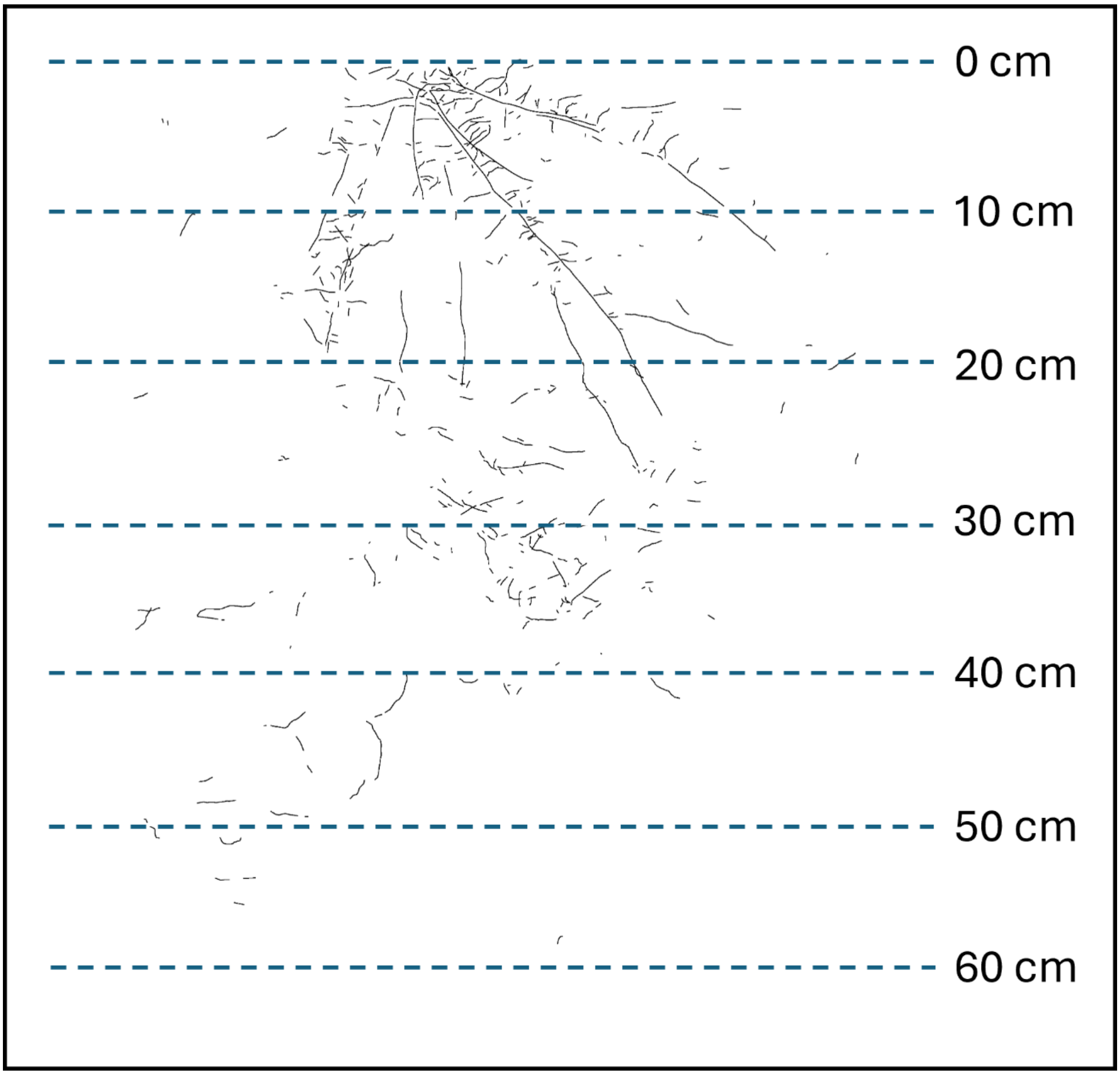

Figure S7 *One example of M. sin roots traced using the SmartRoot plug-in programme for ImageJ (for clarity, in this image, line widths are not shown to scale) as at 31 July.*

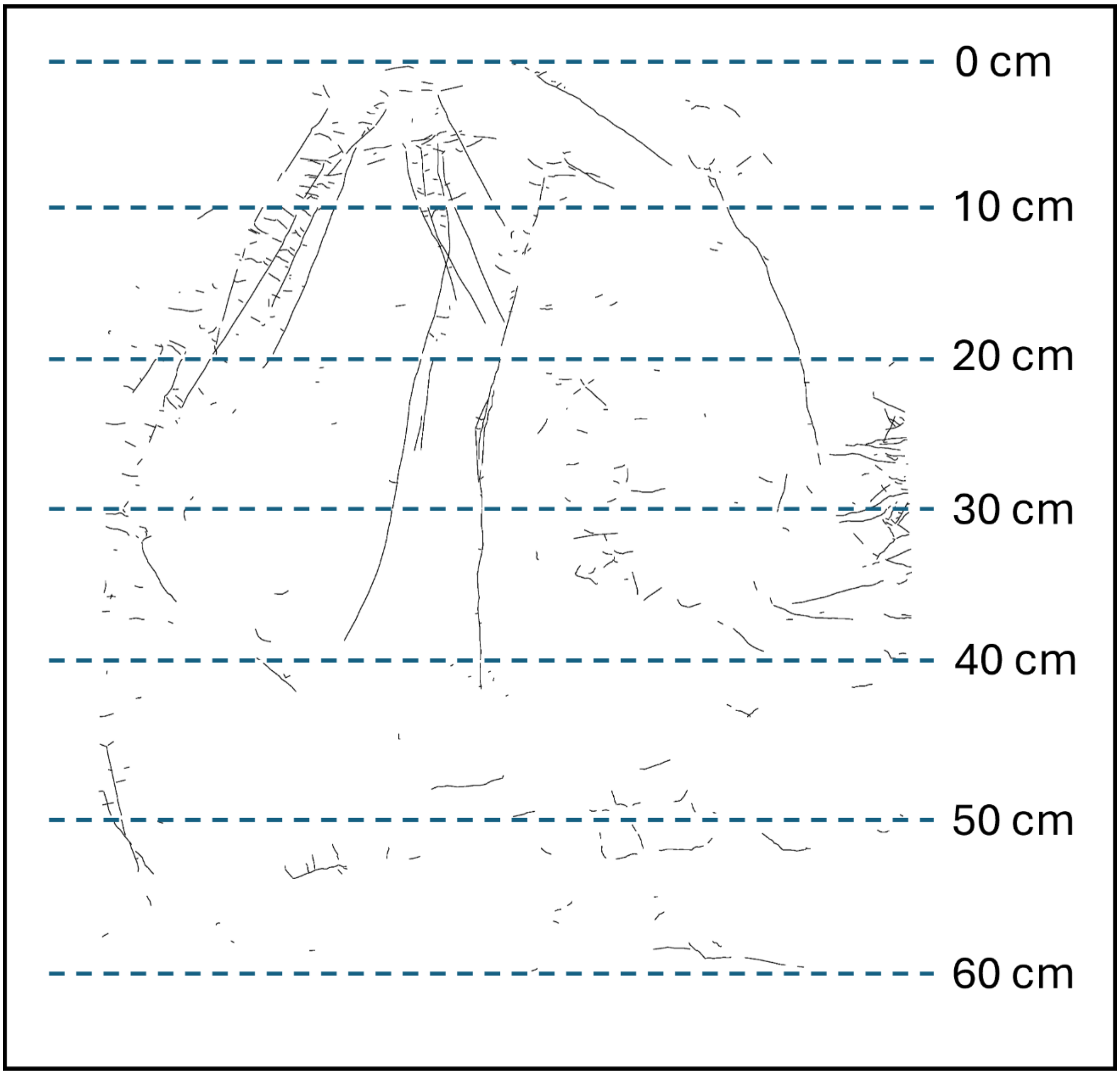

Figure S8 *One example of M×g roots traced using the SmartRoot plug-in programme for ImageJ (for clarity, in this image, line widths are not shown to scale) as at 31 July.*
